# Engineering of OHet72 Nanocrystals into Nanostructured Microparticles for Pulmonary Delivery of a Novel Antitubercular Drug Candidate

**DOI:** 10.3390/pharmaceutics18091164

**Published:** 2026-09-16

**Authors:** Margaret D. Bourlon, Alexa Beathard, Lucila Garcia-Contreras

**Affiliations:** Department of Pharmaceutical Sciences, University of Oklahoma Health Science Center, Oklahoma City, OK 73117, USA; mdbourlon@gmail.com (M.D.B.); alexa-beathard@ou.edu (A.B.)

**Keywords:** tuberculosis, OHet72, dissolution enhancement, spray drying, nanocrystals, nanostructured microparticles, apparent solubility, deposition

## Abstract

**Background/Objectives**: Current treatments for tuberculosis (TB) are complex and associated with severe side effects; thus, drugs with minimal side effects would be desirable as additions to current therapies to combat the global TB epidemic. OHet72 is a novel compound with in vitro activity against *Mycobacterium tuberculosis* but has poor water solubility. As a first step in assessing its potential for TB treatment, the goal of this study was to formulate OHet72 in a powder form that will increase its solubility compared to the unprocessed drug, and when aerosolized, ensure that most of the inhaled dose would be deposited in the alveoli. **Methods**: OHet72 was first formulated into nanocrystals (NCs) and then spray-dried to obtain nanostructured microparticles (nsMPs). The resulting formulations were characterized in vitro, and their rate of dissolution was assessed. **Results**: OHet72 NCs were oblong-shaped with an average width of 145 nm and a length of 255 nm, whereas the resulting nsMPs were spherical, with a geometric diameter of 0.64 μm and a GSD of 1.54. When aerosolized with the Pari LC^®^ Star nebulizer, a suspension of OHet72 NCs in saline had a mass median aerodynamic diameter (MMAD) of 3.15 ± 0.43 μm and a fine particle fraction (FPF, indicating the fraction that will likely be deposited in the alveoli) of 54.63 ± 5.83%. In contrast, OHet72 nsMPs dispersed from the Plastiape RS01 dry powder inhaler (DPI) had a MMAD of 3.80 ± 0.49 μm and an FPF of 37.16 ± 6.67%. The dissolution rate of OHet72 NCs was faster (34.65 ± 5.43%) than that of the unprocessed drug (0.72 ± 0.34%), but the OHet72 in the nsMPs dissolved significantly faster and to a greater extent (57.55 ± 5.60%) than both the unprocessed drug and the NCs. **Conclusions**: Based on their MMAD, GSD and FPF, OHet72 NC and nsMPs appear suitable for effective pulmonary delivery, and based on their apparent solubility and dissolution testing, they would dissolve in the lung environment.

## 1. Introduction

With COVID-19 now largely under control, tuberculosis (TB) is again the leading global cause of death from a single infectious agent, specifically *Mycobacterium tuberculosis* (MTB) [1]. Drug-susceptible TB (DS-TB) is treated with the four first-line anti-TB drugs (rifampicin, isoniazid, pyrazinamide, and ethambutol) [2] and has undesirable side effects. Existing treatments for multidrug-resistant TB (MDR-TB) consist of 4 to 10 anti-TB drugs and are associated with more severe side effects than the first-line therapy [1]. Thus, new drugs with little to no side effects are highly desirable.

Over the last decade, we have discovered that a handful of heteroarotinoid compounds from a novel class of anticancer agents have in vitro activity against DS- and MDR-TB. The lead compound in terms of anticancer activity, SHetA2 [3,4,5,6,7], had a minimum inhibitory concentration (MIC) of 10 μg/mL against DS-TB. Formal toxicity studies found no adverse effects in either rats or dogs after administration of SHetA2 at concentrations 30-fold higher than the effective anticancer dose [8]. Phase I clinical trials that evaluated the efficacy of SHetA2 to treat ovarian, cervical, and endometrial cancers [9,10] have been completed, and a Phase II clinical trial will begin this year. A second compound in the same class, OHet72, was more potent against DS-TB (MTB H37Rv) than SHetA2 with an MIC of 1 μg/mL [11] and was also effective in vitro against three clinical isolates of MDR-TB. However, these heteroarotinoid compounds have low water solubility, which limits their bioavailability (<10%) after oral administration [12].

TB treatment by the pulmonary route has been evaluated for decades [13], and its advantages have been extensively reviewed in the literature [14,15,16,17]. Advantages of the pulmonary route include the possibility of enhancing the dissolution of drugs with poor water solubility due to the presence of surfactants in the lung epithelial lining fluid [18,19,20]; thus, achieving high local concentrations from a pulmonary dose that is significantly lower than an oral dose [21].

As the first step toward our overarching goal of assessing the potential of OHet72 to treat TB via the pulmonary route, we optimized the formulation of OHet72 to maximize its dissolution in the airways and alveoli and subsequent absorption into lung tissue. To this end, OHet72 was prepared as nanocrystals (NCs) and NC size was optimized to increase the rate of drug dissolution in the lung epithelial lining fluid [22]. To optimize their pulmonary delivery, OHet72 NCs were formulated as nanostructured microparticles (nsMPs), using mannitol as the core. The present study describes the optimization of the preparation of OHet72 NCs and nsMPs as well as their physicochemical evaluation.

## 2. Materials and Methods

### 2.1. Materials

OHet72 was synthesized as described previously [23]. Briefly, 4,4-dimethyl-3,4-dihydro-2H-1-benzopyran-6-carboxylic acid was first mixed with 4-hydroxybenzaldehyde in dichloromethane and dicyclohexylcarbodiimide. The resulting aldehyde was condensed with thiosemicarbazide to produce OHet72 (thiosemicarbazone). Solvents (acetone, acetonitrile, methanol, ethanol, chloroform, and dichloromethane) were all HPLC or analytical grade and purchased from Sigma-Aldrich (St. Louis, MO, USA). Deionized water was obtained from a Milli-Q water purification system (Millipore, Burlington, MA, USA). Polyvinyl alcohol (PVA) was purchased from Polysciences Inc. (Warrington, PA, USA), D-mannitol from Sigma-Aldrich, and phosphate-buffered saline (PBS) tablets from Fisher Scientific (Waltham, MA, USA).

### 2.2. Solubility of OHet72

To select a solvent for the controlled crystallization of OHet72, we first assessed OHet72 solubility in various solvents (acetone, acetonitrile, methanol, ethanol, chloroform, dichloromethane, and deionized water) by the gradual addition of a small volume of each solvent to a known mass of unprocessed drug [24]. After each addition, the sample vial was vortexed (VWR, Radnor, PA, USA) and ultrasonicated (Branson 3510, Brookfield, CT, USA) to aid in the dissolution of the compound.

### 2.3. Formulation and Optimization of OHet72 NCs

OHet72 nanocrystals (NCs) were prepared using the bottom-up solvent–antisolvent (S-AS) controlled crystallization method [25,26,27]. A 50 mg/mL solution of OHet72 in acetone was added dropwise into a solution of PVA in a 25 mL round bottom flask. Probe sonication (Fisher Scientific, Waltham, MA, USA) was used to disperse the acetone droplets in the PVA solution, and ultrasonication was used to control the rate of crystal growth after nucleation. Crystallization parameters were optimized to obtain NCs of <200 nm using Design of Experiments software (DoE, Design Expert, Stat-Ease, Inc., Minneapolis, MN, USA). The following parameters were entered into a 2^3^ full factorial design (DoE1): sonication amplitude (50–100%), sonication time (30–60 min), and ultrasonication time (30–60 min). PVA concentration (2% *w*/*v*) and S-AS ratio (1:10) remained constant in this design. A subsequent design, DoE2, was used to further reduce NC size by entering the following manufacturing parameters into a 2^2^ full factorial design: PVA concentration (1–2% *w*/*v*) and S-AS ratio (1:10–1:20). Sonication amplitude (50%), sonication time (60 min), and ultrasonication time (30 min) were the same in DoE2 as those in DoE1.

### 2.4. Formulation and Optimization of OHet72 nsMPs

OHet72 nanostructured microparticles (nsMPs), comprising OHet72 NCs and mannitol (50:50 *w*/*w*), were manufactured using a Buchi-290 mini spray dryer (Buchi, Switzerland) using the high-performance cyclone to increase the yield of small batches. OHet72 NCs were suspended in a solution of mannitol (1:1 OHet72: mannitol, 0.1% total *w*/*v*) under constant stirring (VWR, Radnor, PA, USA) to prevent crystal settling during spray drying. The spray drying parameters were a nitrogen flow rate of 742 L/h, an OHet72/mannitol feed flow rate of 3 mL/min, an aspiration rate of 35 m^3^/h, and an inlet temperature of 120 °C. The proportion (*w*/*w*) of NCs and mannitol, as well as the parameters of spray drying, were previously optimized in our laboratory to enhance the aerosol performance of nsMPs in a dry powder inhaler [28]. Immediately after preparation, the nsMPs were stored in a glass vial, which was tightly closed and placed inside a bag with desiccant. The bag was placed in a desiccator kept at room temperature (20–22 °C, 25–40% relative humidity, depending on the time of the year) until used.

### 2.5. Characterization of OHet72 NCs and nsMPs

#### 2.5.1. Morphology and Geometric Diameter

The morphology and geometric diameter (Dg) of the unprocessed drug, NCs, and nsMPs were determined from scanning electron microscopy (SEM) images (Zeiss Neon 40 EsB, Oberkochen, Germany). For this purpose, the unprocessed drug and the nsMPs (1–3 mg) were deposited directly on double-coated carbon conductive tape (Ted Pella Inc., Redding, CA, USA) fixed to an aluminum stub. OHet72 NCs were suspended in water (1–2 mg/mL) and a small droplet was deposited on a glass slide fixed to the stub using carbon tape, where it was allowed to air dry. All stubs were sputter-coated (Hummer VIO, Anatech Ltd., Sparks, NV, USA ) with gold-palladium (5–6 nm) for imaging. Individual particles from each image were measured using ImageJ software (version 1.53d, National Institutes of Health, Washington, DC, USA). At least 200 particles were measured for each determination. The particle size versus cumulative frequency data was plotted on log probability paper to determine the Dg and its corresponding geometric standard deviation (GSD_Dg_) [6].

#### 2.5.2. Fourier Transform Infrared Spectroscopy (FTIR)

The chemical stability of OHet72 NCs and nsMPs was evaluated using a Nicolet is10 ATR-FTIR with a diamond crystal (ThermoFisher, Waltham, MA, USA). The OHet72 NCs were dried by filtering and drying the suspension on 0.2 µm filter paper. The NCs were then collected from the filter paper into glass vials. Dry powders of unprocessed OHet72, PVA, dried OHet72 NCs, mannitol, and OHet72 nsMPs were placed as a thin layer (1 µm) covering the crystal for analysis. FTIR spectra were collected from 400 to 4000 cm^−1^ with a resolution of 0.5 cm^−1^ and analyzed using OMNIC^®^ Spectra software (ThermoFisher, Waltham, MA, USA).

#### 2.5.3. Differential Scanning Calorimetry (DSC)

The solid-state properties of unprocessed OHet72, NCs, and nsMPs were assessed using a DSC instrument (DSC-60, Shimadzu, Kyoto, Japan). Approximately 3–5 mg of unprocessed OHet72, PVA, dried OHet72 NCs, mannitol, and OHet72 nsMPs were loaded each into aluminum pans, which were sealed by crimping the edges. The pans were heated from 50 to 250 °C at a rate of 10 °C/min under a continuous flow of nitrogen (N_2_) gas at 30 mL/min. The heat flow rate was normalized using an empty pan as a control, and the endothermic and exothermic peaks were analyzed using ta60 software (version 2.21, Shimadzu, Kyoto, Japan).

#### 2.5.4. X-Ray Diffraction (XRD)

The crystallinity of unprocessed OHet72, PVA, dried NCs, mannitol, and a physical mixture of NCs and mannitol was evaluated by powder XRD using a Rigaku Ultima IV X-ray diffractometer (Woodlands, TX, USA). Cu-K-alpha radiation was generated at a voltage of 40 kV and a current of 44 mA. Diffraction patterns were collected with a scintillation detector using the 2Θ method with a 0.02 step size for 2 s between 5 and 80°. Alternatively, OHet72 nsMPs were evaluated using a Rigaku SmarLab X-ray diffractometer (Woodlands, TX, USA), with the same methods and a smaller step size of 0.01.

#### 2.5.5. Volume Diameter (Dv)

The volume diameter (Dv) of nsMPs in dry form and the corresponding standard deviation (GSD_Dv_) were determined using a HELOS laser diffraction system (Sympatec Inc., Lawrenceville, NJ, USA) and the dry dispersion RODOS unit. The RODOS dispersion pressure was varied (0.5–3.0 bar) to determine its influence on the resulting Dv and GSD_Dv_. In addition, the Dv of NCs suspended in saline (wet aerosol) was determined using the HELOS cuvette accessory, whereas the INHALER accessory was used to measure the Dv of droplets generated from NCs in suspension using a nebulizer (Pari LC Star, Midlothian, VA, USA). All HELOS measurements were obtained with a time base of 5 milliseconds, and detection was triggered when the optical concentration in the measuring zone exceeded 0.5%.

#### 2.5.6. OHet72 Drug Loading in nsMPs

Drug loading of OHet72 in the nsMPs was determined by dissolving a known mass of nsMPs in an equal volume (i.e., 1 mg in 1 mL) of 65:35 acetonitrile:water. OHet72 concentrations were determined using an Alliance HPLC system (model e2698, Waters, Milford, MA, USA) consisting of an X-Bridge peptide BEH C18, 150 A, 3.5 µm column (150 mm × 4.6 mm), a guard column (X-Bridge BEH C18, 3.5 µm, 3.9 mm × 5 mm) and a UV detector (model 2489, Waters, Milford, MA, USA) set at 315 nm. The mobile phase consisted of an isocratic mixture of acetonitrile:water (65:35 *v*/*v*) at a flow rate of 0.5 mL/min. Each injection was 20 µL, and the total run time was 10 min. OHet72 had a retention time of 7.1 min. The limit of detection (LoD) was 0.025 µg/mL, and the limit of quantitation (LoQ) was 0.050 µg/mL. Drug loading was determined according to the equation:

$$Drug\;Loading=\left(\frac{Actual\;drug\;mass}{Theoretical\;drug\;mass}\right)*100$$

#### 2.5.7. Aerosol Performance

To predict the site of deposition of the inhaled OHet72 formulations, the aerosol performance was evaluated using a next generation impactor (NGI, Westech, Chelmsford, UK). The NGI comprised an induction port, seven stages with decreasing pore diameters, and a micro-orifice collector (MOC). A vacuum pump (Westech VTE 6) was used in conjunction with the NGI to simulate inspiration. For OHet72 nsMPs aerosolized from a Plastiape RS01 Monodose DPI, nsMPs (20 ± 1 mg) were weighed in a size 3 hydroxypropyl methylcellulose capsule (Capsugel, Morristown, NY, USA). The amount of nsMPs was selected so that it would comfortably fit in the capsule without compacting the powder. The capsule was placed into the Plastiape DPI and the device was attached to the induction port using a custom adaptor. After actuation of the DPI, the NGI pump was operated for 5 s at a flow rate of 60 L/min. OHet72 NCs in a saline suspension were aerosolized using a Pari LC Star nebulizer with a Vios Pro pump. The suspension was loaded into the reservoir of the nebulizer. The nebulizer was fixed to the induction port. The nebulizer and the NGI pump (at a 15 L/min flow rate) were activated simultaneously and ran for 5 min. After each experiment, the induction port and collection cups for each stage were rinsed with a 65:35 ratio of acetonitrile and water to collect the deposited particles. The OHet72 content was analyzed by HPLC.

Aerosol performance was evaluated in terms of mass median aerodynamic diameter (MMAD), geometric standard deviation (GSD_MMAD_), emitted dose (ED), and fine particle fraction (FPF). The MMAD was calculated as described in the United States Pharmacopeia, chapter <601>, and the GSD_MMAD_ was calculated in the same manner as GSD_Dg_ and GSD_Dv_. The ED was calculated as:

$$Emitted\;Dose=\;\left(\frac{OHet72\;mass\;recovered\;from\;NGI\;\left(mg\right)}{OHet72\;mass\;loaded\;into\;device\;\left(mg\right)}\right)*100$$

The FPF was calculated as the percentage of particle mass recovered below 4.46 µm in diameter, represented as particles recovered from stages 4–7 and the MOC for aerosols generated by the nebulizer and particles recovered from stages 3–7 and the MOC for aerosols generated by the DPI. The Impactor Sized Mass (ISM) was calculated by adding the mass of OHet72 recovered from all stages and the MOC [29].

### 2.6. Apparent Maximum Solubility in Simulated Lung Fluid

The apparent solubility of the unprocessed drug, OHet72 NCs, and nsMPs was determined in simulated lung fluid (SLF), consisting of PBS with 0.05% (*w*/*v*) sodium dodecyl sulfate (SDS) at a pH of 7.4 [30]. Excess amounts of unprocessed OHet72, NCs, and nsMPs were added to screw cap vials containing 5 mL of SLF, and the vials were incubated for 24 h in a water bath shaker (VWR, Radnor, PA, USA) at 37 °C and 100 RPM. At predetermined time points, 500 μL aliquots were withdrawn from each sample and replaced with equal volumes of fresh SLF.

Previously, our laboratory used filtration to separate solids from dissolved drug, but since the size of some NCs was smaller than the pore size of the filter, we developed a separation method using sequential ultracentrifugation, based on previously published methods [31,32]. Specifically, each sample was first centrifuged for 10 min at 16,100 RCF (Eppendorf 5415 R, Hamburg, Germany). The supernatant was collected and centrifuged a second time (Eppendorf 5424, Hamburg, Germany) using the same conditions. The supernatant collected after the second centrifugation was diluted with an equal volume of acetonitrile, and the OHet72 concentration was determined by HPLC.

### 2.7. In Vitro Dissolution of NCs and nsMPs

The dissolution of OHet72 NCs and the respirable fraction of OHet72 nsMPs was evaluated using an apparatus developed by our laboratory [6]. Our apparatus combines aspects of similar apparatuses described by Davies et al. [33] and Wang et al. [34], but has additional features to simulate the small volume available for drug dissolution in the alveolar region of the human lungs while maintaining sink conditions. NCs were concentrated in saline suspension and directly used in the dissolution experiments. The respirable fraction of nsMPs (size range 2.82–4.46 μm) was separated as previously described [6]. Briefly, a glass fiber filter (WG653-47; Westech Scientific Inc., GA, USA) was placed on the collection cup of stage 3 of the NGI, and then OHet72 nsMPs (20 ± 1 mg) were weighed in a size 3 hydroxypropyl methylcellulose capsule (Capsugel, Morristown, NY, USA). The capsules were loaded into the Plastiape DPI, which was attached to the NGI. After actuation of the device, the NGI was operated for 60 s at a flow rate of 60 L/min to maximize the deposition of particles on the glass filter. The glass fiber filter with the NCs or nsMPs was then transferred to our custom-made dissolution apparatus [6]. The dissolution apparatus consisted of a jacketed, 20 mL Franz diffusion cell (PermeGear, Inc., Hellertown, PA, USA) used as a flow through cell, and a dual-channel pump (Cole-Parmer, Vernon Hills, IL USA). The dual pump was simultaneously used to deliver SLF into the donor compartment and to circulate heated water into the jacket of the diffusion cell to maintain the temperature at 37 °C in the receptor compartment. The glass fiber paper holding the OHet72 NCs or nsMPs was sandwiched between two additional glass fiber filters and placed in the Franz cell between the donor and recipient compartments. The SLF was pumped onto the glass fiber paper at a flow rate of 2 mL/min, and samples were collected from the receiving compartment every 5 min for 5 h. Because the smallest NCs were able to go through the filter, each sample was centrifuged as described in Section 2.6 to separate the undissolved drug that passed through the glass fiber filter. The undissolved and dissolved drug at each timepoint, as well as the amount of drug remaining in the filter at the end of the experiment, were quantified by HPLC, and each experiment was performed in triplicate.

### 2.8. Statistical Analysis

The values of the NCs and nsMPs sizes and distribution responses were analyzed with the DoE software. The absolute values of the effect were initially displayed in a half-normal probability plot, and the magnitude of each effect was confirmed in a Pareto chart. The effects with the largest magnitude were then selected and analyzed with an ANOVA, and the equations quantifying the outcome of each variable were built using the effects considered significant. The significance of all other results was evaluated using GraphPad Prism software (version 10.0.2; Dotmatics, Boston, MA, USA). A *p*-value less than 0.05 was considered significant.

## 3. Results

### 3.1. OHet72 Solubility

OHet72 had the highest solubility in acetone (>30 mg/mL), followed by acetonitrile (9.94 mg/mL) and dichloromethane (6.50 mg/mL), while solubility was either low (<1.00 mg/mL) or OHet72 was insoluble in all other solvents tested.

### 3.2. Characterization of OHet72 NCs and nsMPs

#### 3.2.1. Size and Morphology

**Figure 1:**
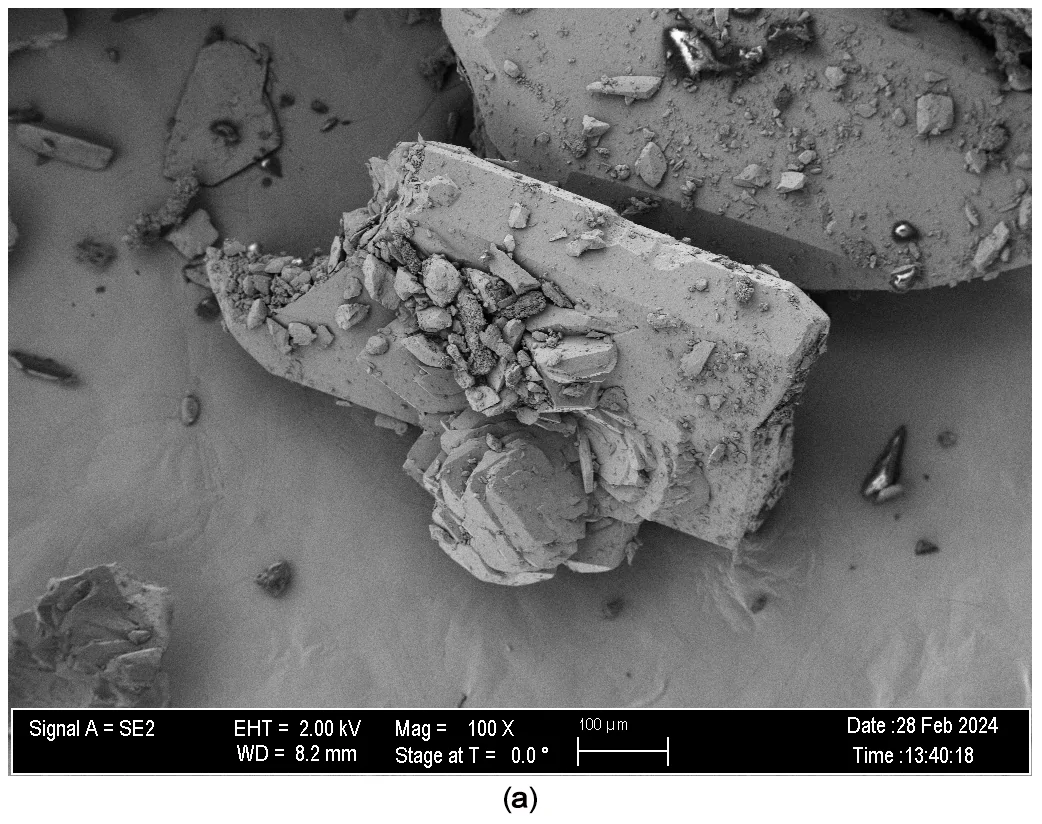

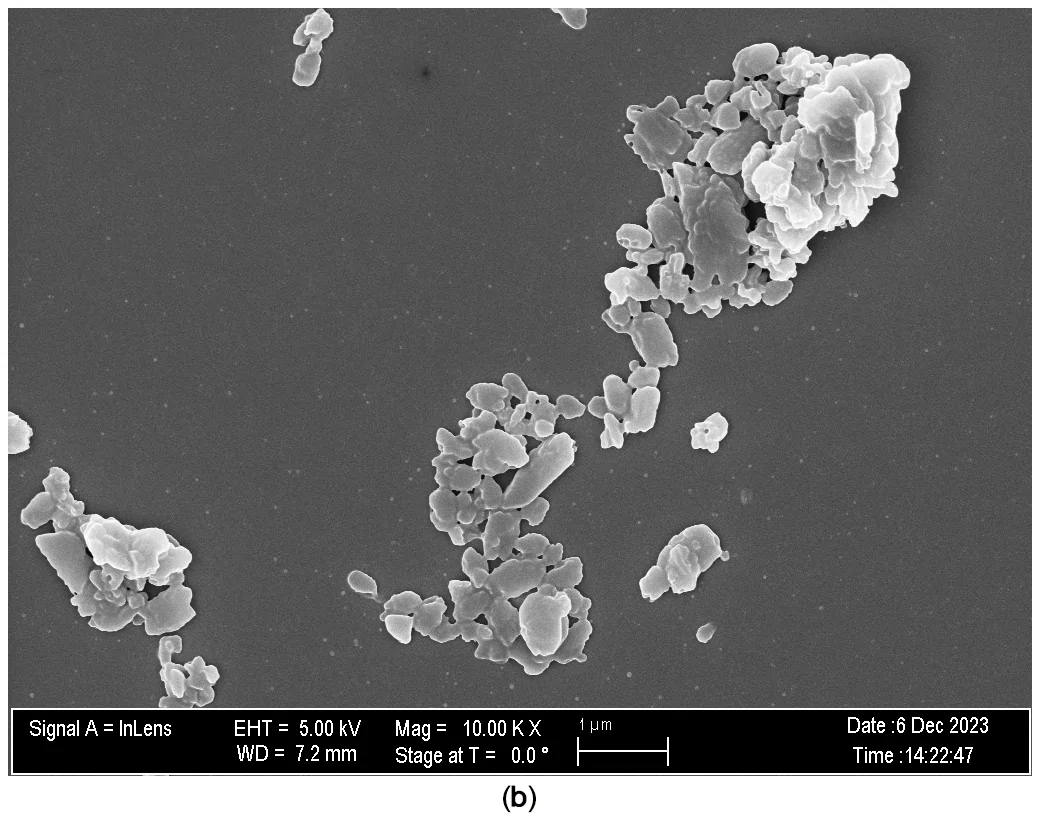
Representative SEM images of (**a**) unprocessed OHet72, and (**b**) OHet72 NCs. Scale bars are shown on each individual image.

The experimental parameters that were entered into DoE1 to optimize the size of NCs and their resulting size and distribution are listed in Table 1. SEM images (Figure 1b) revealed that the NCs were oblong-shaped; thus, their width and length were determined. NC width ranged from 480 to 1500 nm with GSD from 1.44 to 3.02, while their length ranged from 1050 to 2000 nm with GSD from 1.74 to 3.00.

**Figure 2:**
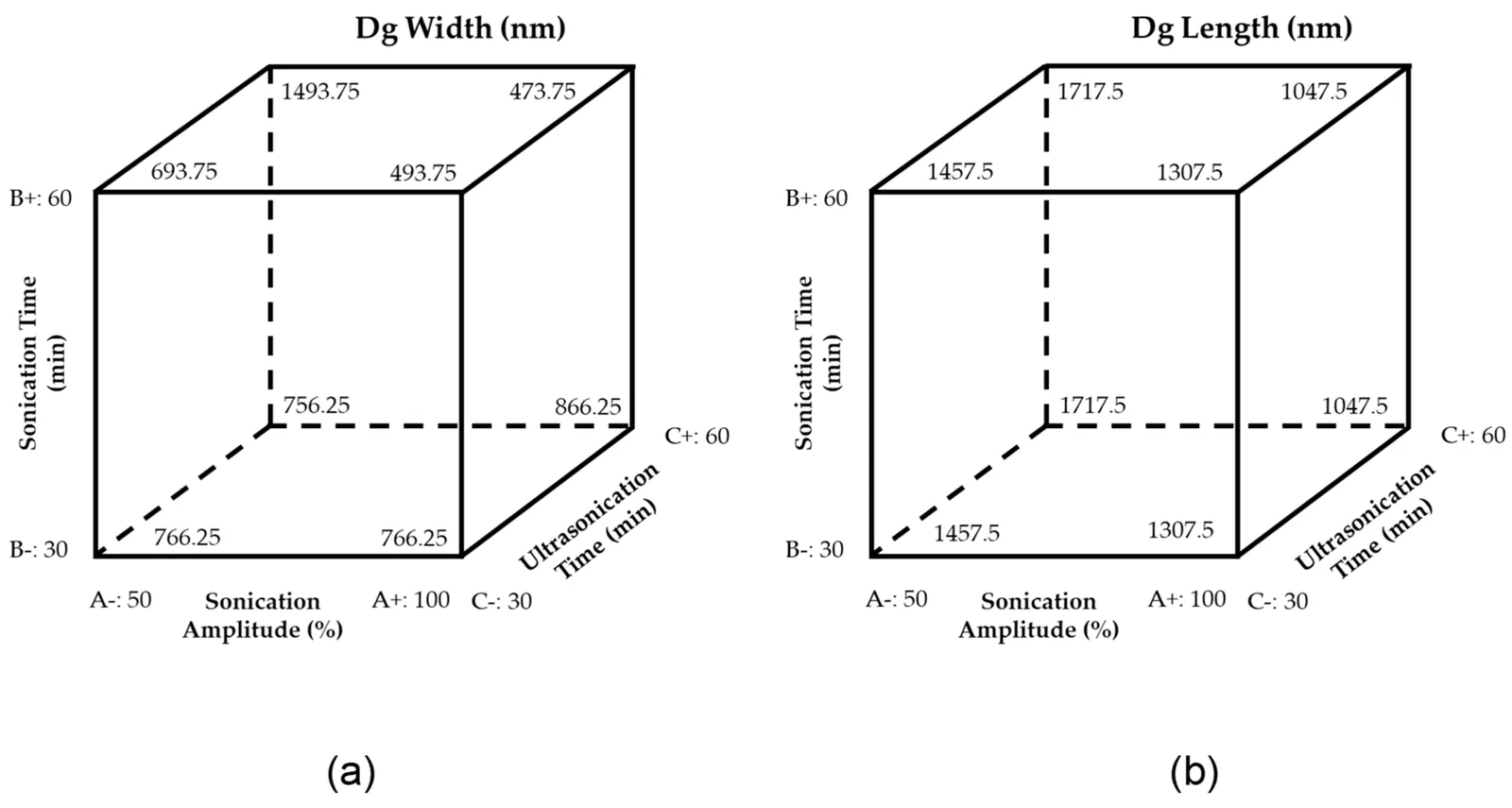
Models generated by the analysis of DoE1: cube plots showing the influence of the experimental factors employed in the preparation of NCs on (**a**) NC width and (**b**) NC length.

The influence of the preparation parameters on the width and length of NCs is shown in Figure 2. Statistical analysis of these parameters determined that the interaction between sonication amplitude (A) and sonication time (B) had the greatest influence on NC width (AB, *p* = 0.0239) followed by ultrasonication time (C, *p* = 0.342) and the interaction between the three parameters (ABC, *p* = 0.0365). At short sonication times (B = 30 min) the variation in sonication amplitude did not influence NC width (766 nm at A= 50% or 100%), but at longer sonication times (B = 60 min) the width of the NC decreased from 693 nm to 493 nm when increasing the amplitude from 50% to 100%. Moreover, when ultrasonication time (C) increased from 30 to 60 min, the width of the NCs decreased from 493 nm to 473 nm. The narrowest NCs were obtained with an amplitude of 100%, a sonication time of 60 min and an ultrasonication time of 60 min.

Statistical analysis of the GSD data for the width of NCs (GDS_W_) also indicated that the interaction between sonication amplitude and sonication time had the greatest influence on NC GDS_W_ (AB, *p* = 0.0427). However, the overall model for GDS_W_ was not statistically significant because of the conflicting relationship between the interaction terms: NCs with the narrowest GDS_W_ were prepared at an amplitude of 100%, a sonication time of 30 min and an ultrasonication time of 30 min or with an amplitude of 50%, a sonication time of 60 min and an ultrasonication time of 30 min.

In contrast, sonication amplitude (A) was the only parameter that significantly influenced the length of NCs (*p* = 0.0335, Figure 2b) with shorter NCs obtained at an amplitude of 100% regardless of the sonication or ultrasonication time. However, statistical analysis of the GSD data for the length of NCs (GDS_L_) indicated that the interaction between the three parameters (ABC) most significantly influenced the GDS_L_ of NCs (*p* = 0.0136), followed by the interaction between sonication time and ultrasonication time (BC, *p* = 0.032). NCs with the narrowest GDS_L_ were prepared at an amplitude of 50%, a sonication time of 30 min and an ultrasonication time of 60 min or with an amplitude of 50%, a sonication time of 60 min and an ultrasonication time of 30 min.

Based on these results, DoE2 was designed to further reduce NC size by investigating the effects of PVA concentration and the S-AS ratio on the crystallization process. Table 2 lists the parameter values entered into DoE2 as well as the resulting NC sizes and GDS. The NC width ranged from 145 to 285 nm, and the GSD of the width ranged from 1.67 to 1.93, whereas the length of the NCs ranged from 255 to 595 nm, and the GSD of the length ranged from 1.71 to 2.21.

**Figure 3:**
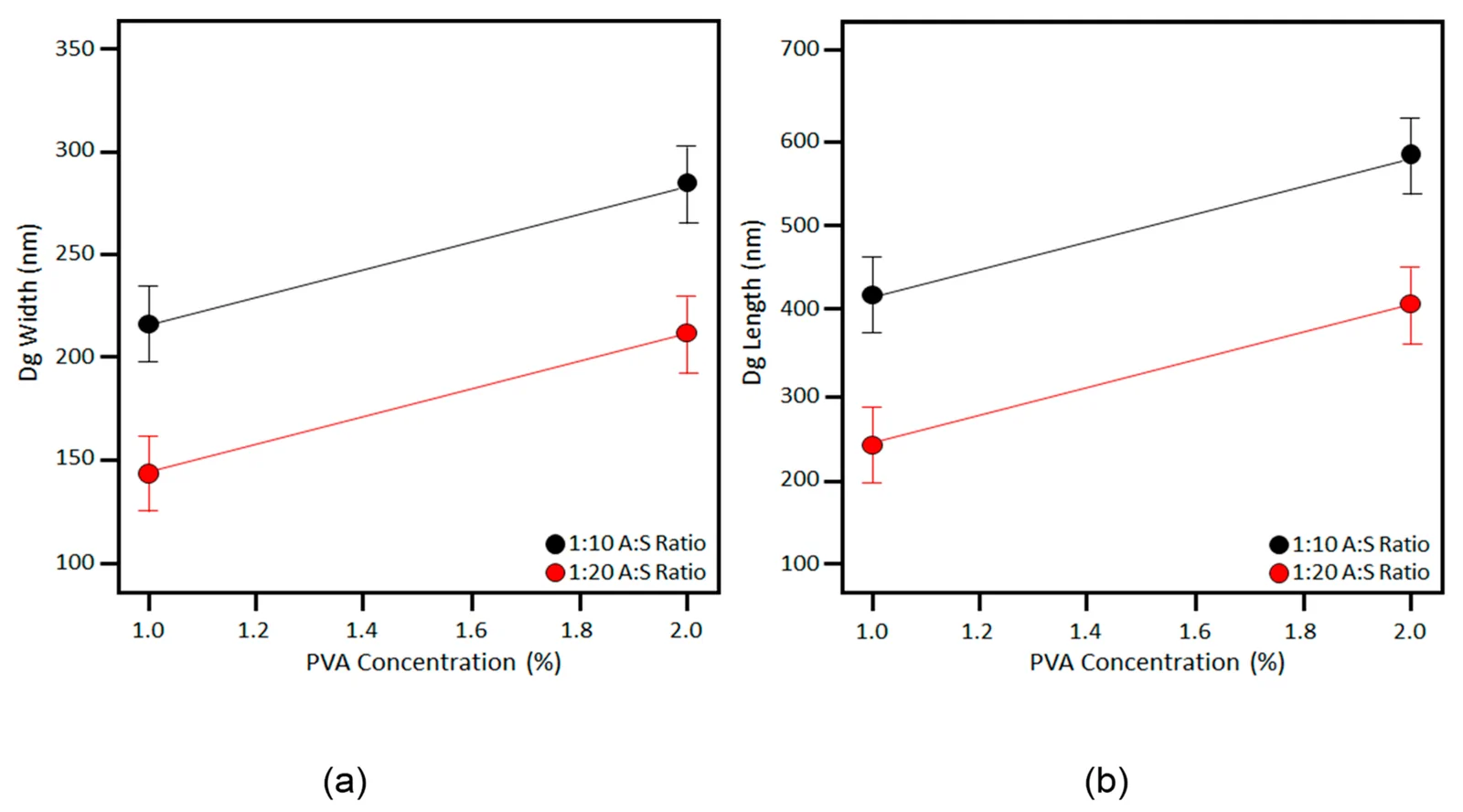
Models generated by the analysis of DoE2: interaction plot showing the influence of the experimental factors employed in the preparation of NCs (**a**) width of the geometric diameter (DgWidth) and (**b**) length of the geometric diameter (DgLength).

Statistical analysis of the results of DoE2 indicated that both the PVA concentration and the S-AS ratio significantly influenced the width of NCs (*p* = 0.0236 and *p* = 0.0219, respectively). Figure 3a shows that the width of NCs could be reduced by decreasing PVA concentration from 2% to 1% and increasing the S-AS ratio from 1:10 to 1:20. Statistical analysis of GDS_W_ in DoE2 also indicated that the interaction between sonication amplitude and sonication time was the factor that most influenced the GDS_W_ of NCs (AB, *p* = 0.0121). NCs with the narrowest GDS_W_ were prepared with 1% PVA and an S-AS ratio of 1:10, or with 2% PVA and an S-AS ratio of 1:20.

Likewise, both the PVA concentration and the S-AS ratio influenced the length of NCs (*p* = 0.0560 and *p* = 0.0560, respectively). Figure 3b shows that the length of NCs could be reduced by decreasing PVA concentration from 2% to 1% and increasing the S-AS ratio from 1:10 to 1:20. However, statistical analysis of the GDS_L_ data for DoE2 indicated that the main parameter controlling the GDS_L_ of NCs was the S-AS ratio (*p* = 0.0635), followed by the PVA concentration (*p* = 0.0948). NCs with the narrowest GDS_L_ in DoE2 were prepared with an S-AS ratio of 1:20 and 2% PVA (GSD = 1.71) or an S-AS ratio of 1:20 and 1% PVA (GSD = 1.94).

Taken together, DoE1 and DoE2 (Figure 2 and Figure 3) indicated that the overall smallest NCs were obtained with the following optimized manufacturing parameters: 50% sonication amplitude, 60 min sonication time, 30 min ultrasonication time, 1% PVA concentration, and 1:20 S-AS ratio.

**Figure 4:**
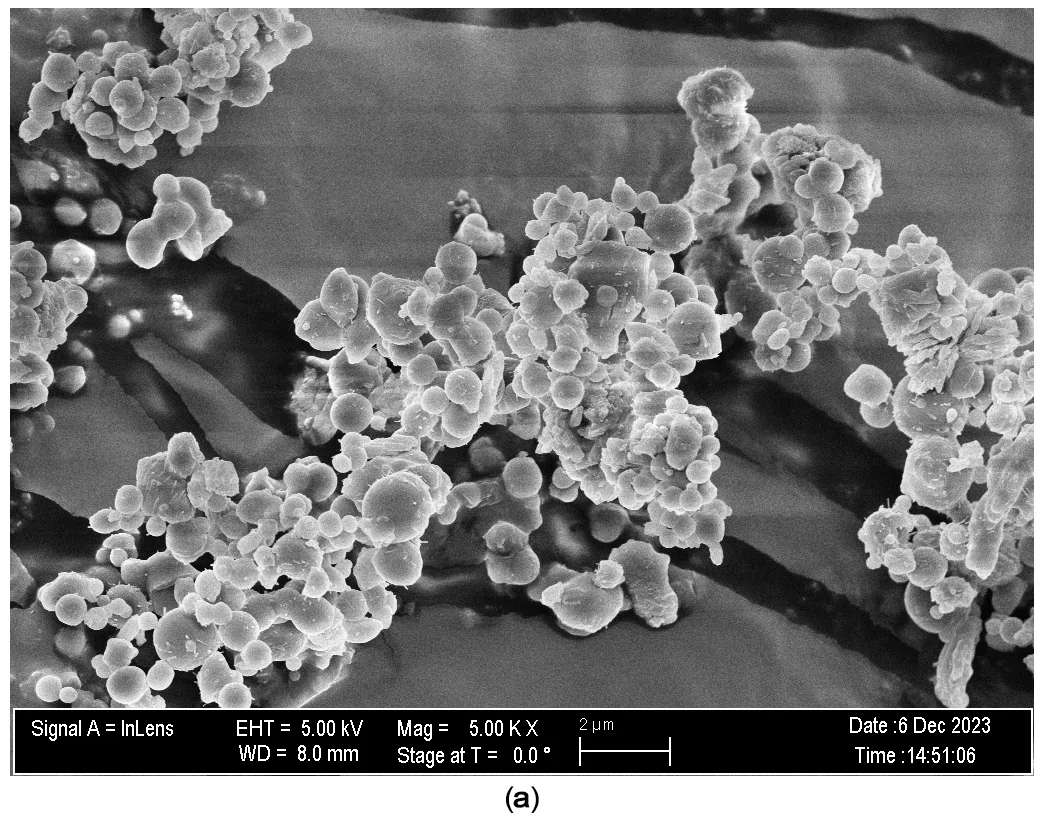

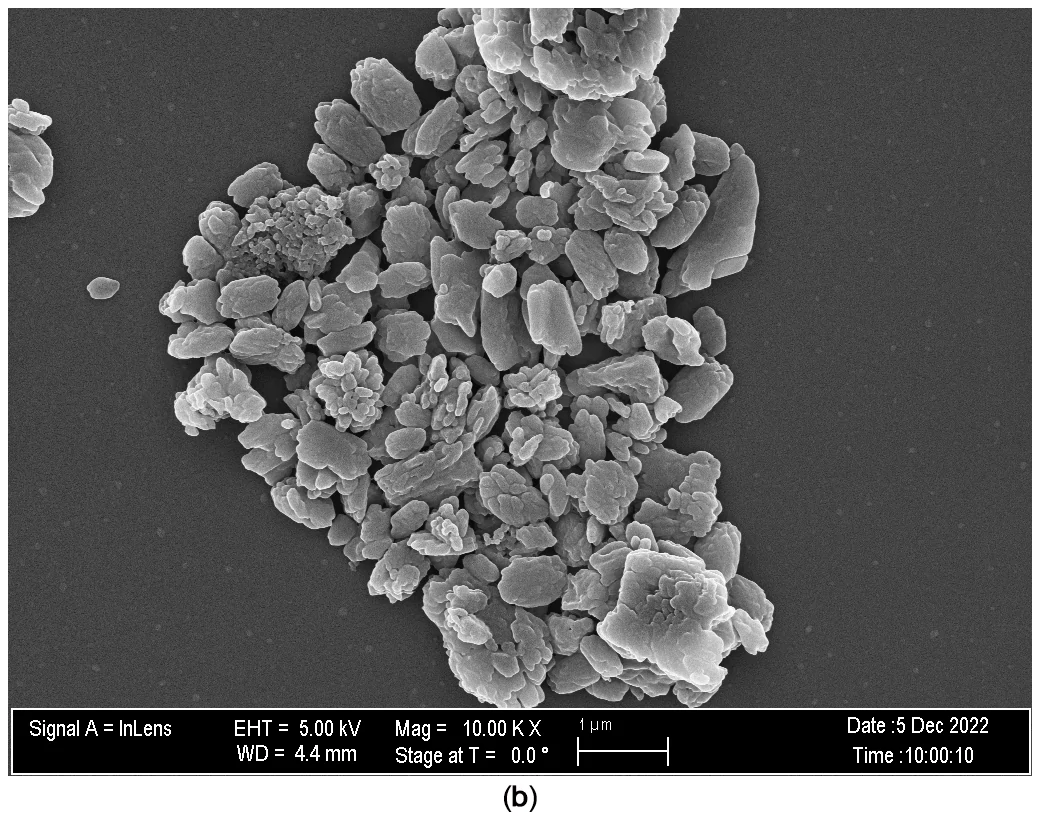
Representative SEM images of (**a**) OHet72 nsMPs, and (**b**) NCs isolated from spray-dried nsMPs. Scale bars are shown on each individual image.

To facilitate pulmonary administration and enhance the efficiency of delivery to the alveoli, the optimized OHet72 NCs were suspended in a mannitol solution and spray-dried to prepare nsMPs. The resulting nsMPs had a Dg of 0.64 µm and a GSD of 1.54 (Figure 4a). After placing the nsMPs in water, it was discovered that the diameter of the NCs increased slightly during spray drying (Figure 4b) to a length of 280 nm and a width of 160 nm (GSD = 1.57 and 1.52, respectively).

#### 3.2.2. Fourier Transform Infrared Spectroscopy (FTIR)

**Figure 5:**
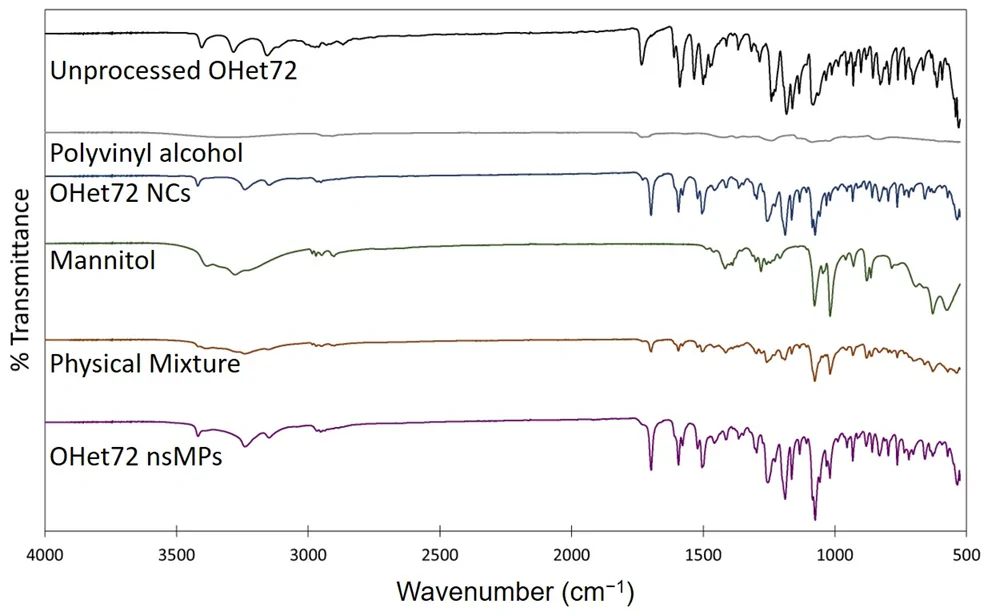
FTIR spectra of unprocessed OHet72, OHet72 NCs, OHet72 nsMPs and their individual ingredients.

The chemical stability of OHet72 after controlled crystallization and spray drying, evaluated by FTIR, is shown in Figure 5. The spectra for the unprocessed OHet72, NCs, and nsMPs had the same characteristic peaks, indicating that the process did not alter the chemical composition of OHet72.

#### 3.2.3. Differential Scanning Calorimetry (DSC)

**Figure 6:**
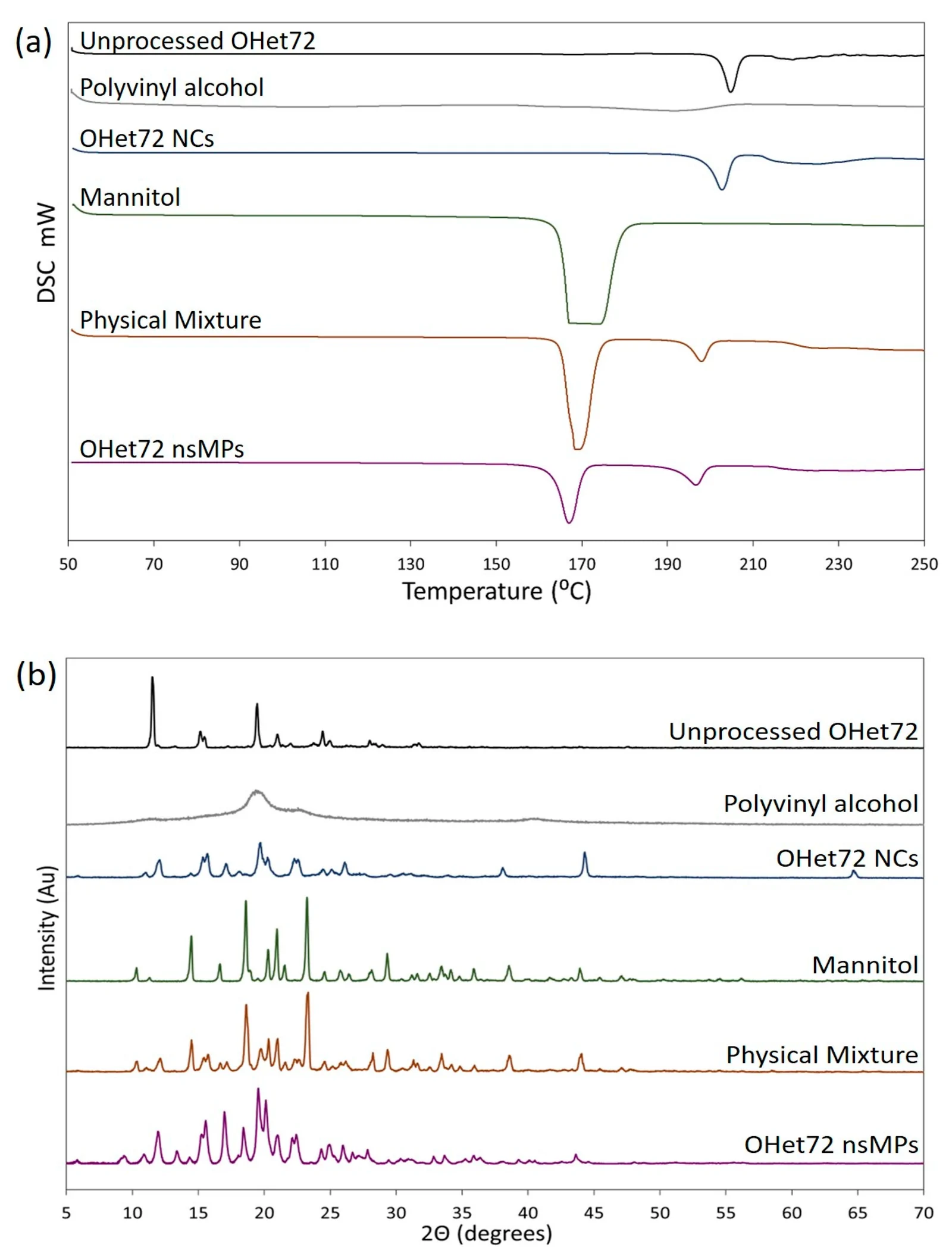
Characterization of unprocessed OHet72, PVA, and OHet72 NCs by (**a**) DSC and (**b**) XRD.

Thermograms comparing the thermal stability of OHet72 NCs and nsMPs to that of the unprocessed drug are shown in Figure 6a. Unprocessed OHet72 had a sharp endothermic peak at 205 °C, corresponding to its melting point. OHet72 NCs, the physical mixture of NCs and mannitol, and the nsMPs had a sharp endothermic peak around the same temperature. PVA had shallow endothermic peaks at 85 and 191 °C, corresponding to its glass transition and melting temperatures, respectively [35]. Mannitol has a broad endothermic peak at 170 °C, corresponding to its melting temperature. This peak is also observed in the physical mixture of OHet72 NCs and mannitol as well as in the nsMPs.

#### 3.2.4. X-Ray Diffraction (XRD)

The diffractograms comparing the crystallinity of OHet72 NCs with that of the unprocessed drug are shown in Figure 6b. As expected, unprocessed OHet72 was crystalline with several sharp peaks observed at 11.5°, 15.4°, 19.5°, 21.1°, and 24.5° 2Θ. Conversely, PVA was amorphous, as indicated by the “halo” effect in the diffractogram and the lack of sharp peaks. OHet72 NCs were crystalline with peaks similar to those of the unprocessed drug, as well as additional peaks at 17.2°, 22.7°, 26.2°, 38.2°, 44.4°, and 65.0° 2Θ.

#### 3.2.5. Volume Diameter (Dv)

The volume diameter of OHet72 nsMPs was initially determined at a dispersion pressure of 0.5 bar, resulting in a bimodal distribution and a Dv and GSD_Dv_ of 2.68 ± 0.04 µm and 1.39 ± 0.28, respectively. Increasing the dispersion pressure to 3.0 bar resulted in a normal distribution around an average Dv of 2.52 ± 0.01 and GSD of 1.14 ± 0.001.

#### 3.2.6. Drug Content

The theoretical drug content for the formulation was 50% OHet72 *w*/*w*. The actual drug content of OHet72 in nsMPs was determined to be 52.51 ± 4.96% *w*/*w*, meaning that the drug loading efficiency of the nsMPs was 105.02 ± 9.92%.

#### 3.2.7. Aerosol Performance

**Figure 7:**
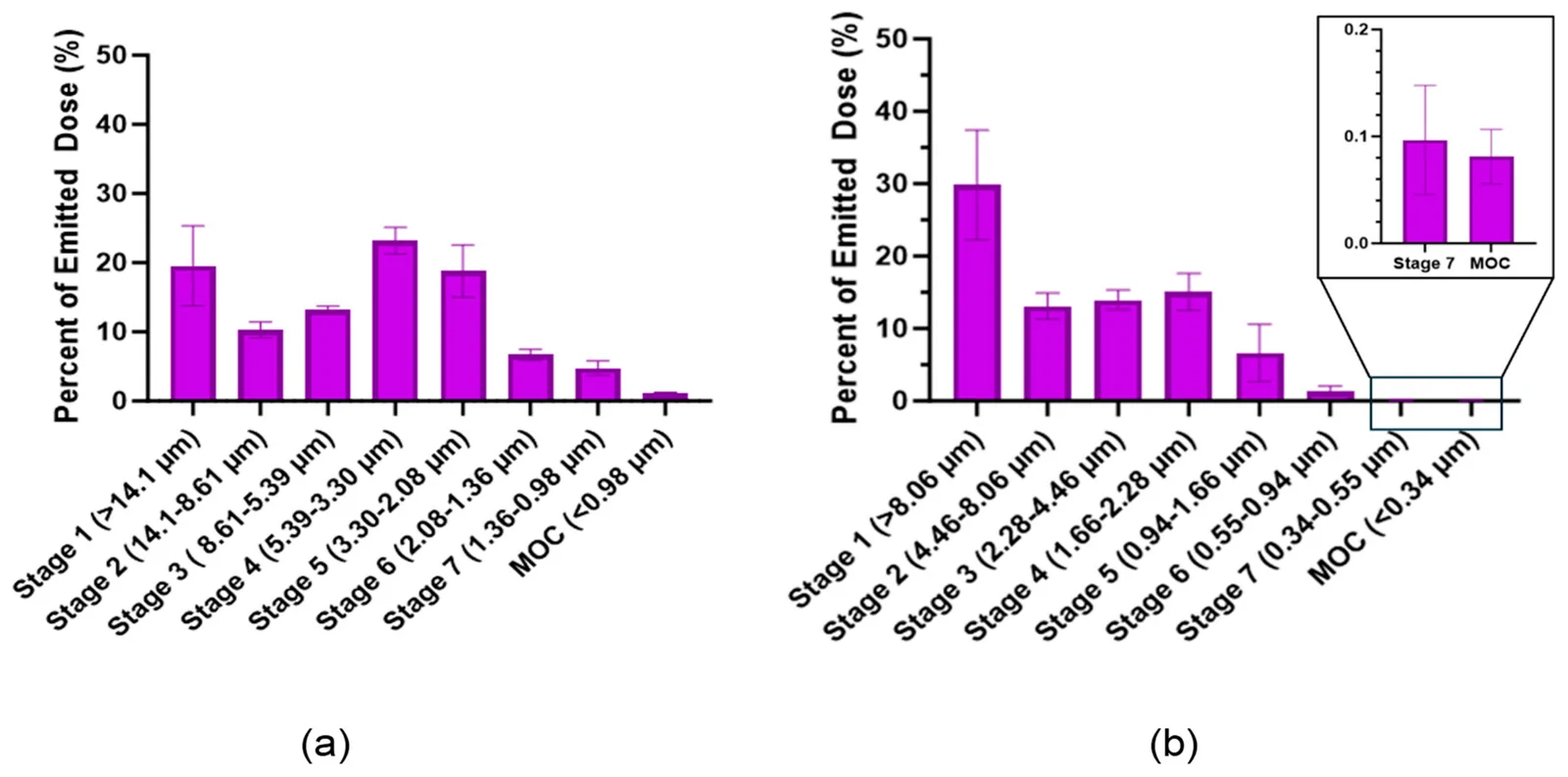
OHet72 deposition pattern in the collection cups of the NGI after aerosolization of NCs in suspension from the Pari LC Star nebulizer (**a**) or dispersion of the nsMPs from the Plastiape RS01 DPI (**b**) (mean ± standard deviation, n = 3).

Figure 7 shows the deposition of OHet72 NCs (7a) and nsMPs (7b) emitted from a nebulizer and a DPI, respectively, across the stages of the NGI. Due to the lower flow rate used when testing the nebulizer compared to the DPI (15 vs. 60 L/min, respectively), the cutoff diameters for each stage are larger for the nebulizer experiments.

Based on the particle deposition, aerosols generated by the nebulizer from the NC suspension had an emitted dose of 1.26 ± 0.47% over 5 min of the dose loaded in the nebulizer, corresponding to an ISM of 0.189 ± 0.071 mg. The aerosol droplets had an MMAD of 3.15 ± 0.43 µm (GSD = 2.81 ± 0.18) and an FPF_<4.6_ of 54.63 ± 5.83%. In comparison, aerosols generated by the DPI from the nsMPs had an emitted dose of 39.60 ± 6.46% of the loaded dose in the DPI, corresponding to an ISM of 3.20 ± 0.51 mg. The dry aerosol particles had an MMAD of 3.80 ± 0.49 µm (GSD = 2.60 ± 0.32), and an FPF_<4.6_ of 37.16 ± 6.67%.

### 3.3. Apparent Solubility

**Figure 8:**
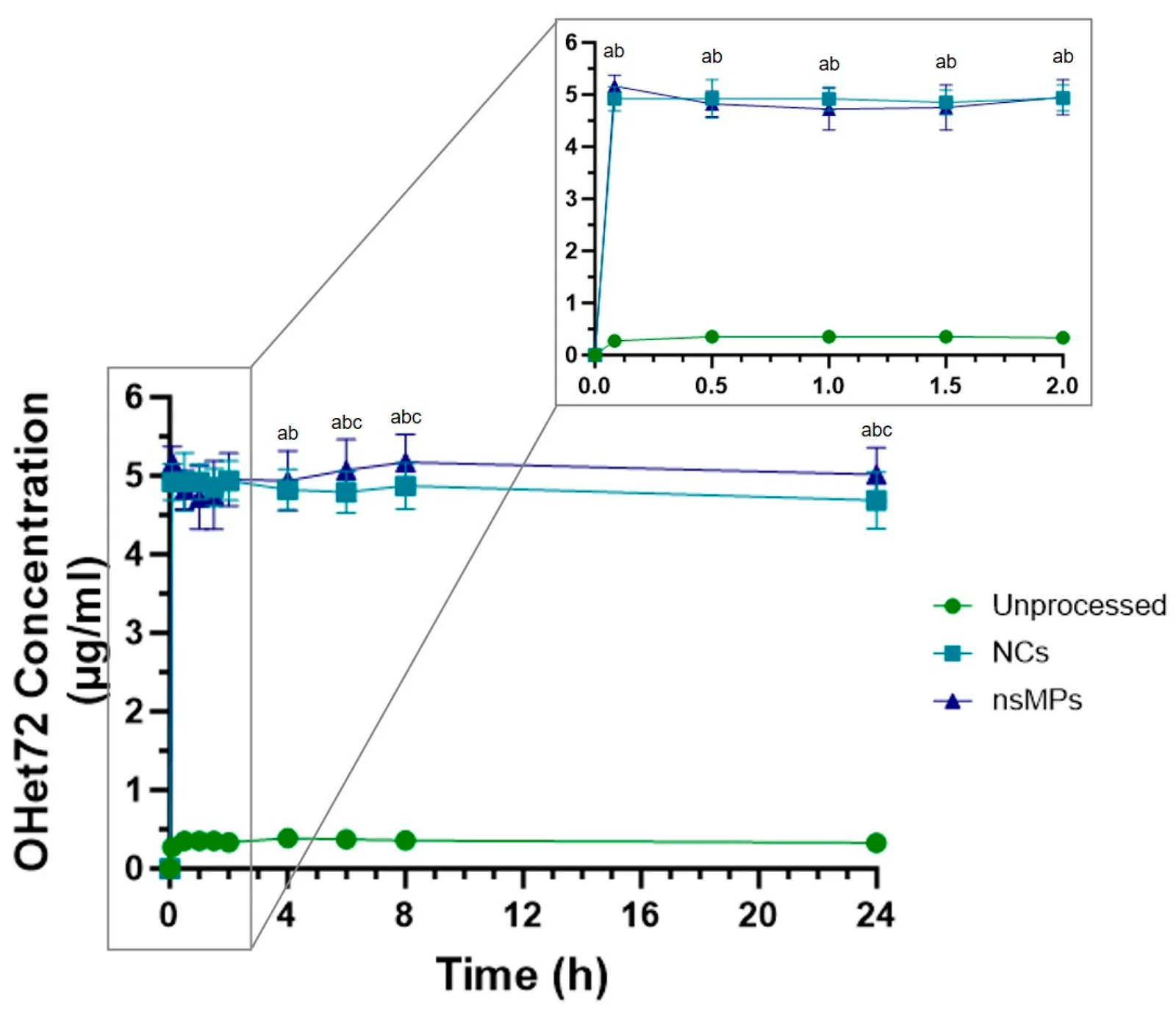
Apparent maximum solubility of OHet72 from unprocessed OHet72, OHet72 NCs, and OHet72 nsMPs in SLF (mean ± standard deviation, n = 3). Letters indicate significant differences between nsMPs and unprocessed (^a^ *p* < 0.0001), NCs and unprocessed (^b^ *p* < 0.0001) or nsMPs and NCs (^c^ *p* < 0.05), determined by two-way ANOVA with multiple comparisons.

Figure 8 shows the apparent solubility of unprocessed OHet72, NCs, and nsMPs in SLF at 37 °C. The NC and nsMP formulations had significantly higher apparent solubility than the unprocessed drug (*p* < 0.0001). Compared to the apparent solubility of the unprocessed drug (0.33 ± 0.09 µg/mL), the apparent solubility of the NCs (4.69 ± 0.39 µg/mL) and nsMPs (5.02 ± 0.32 µg/mL) was increased by 14 and 15-fold, respectively. Starting at 6 h, the apparent solubility of the nsMPs was also significantly higher than that of the NCs (*p* < 0.05).

### 3.4. Dissolution

**Figure 9:**
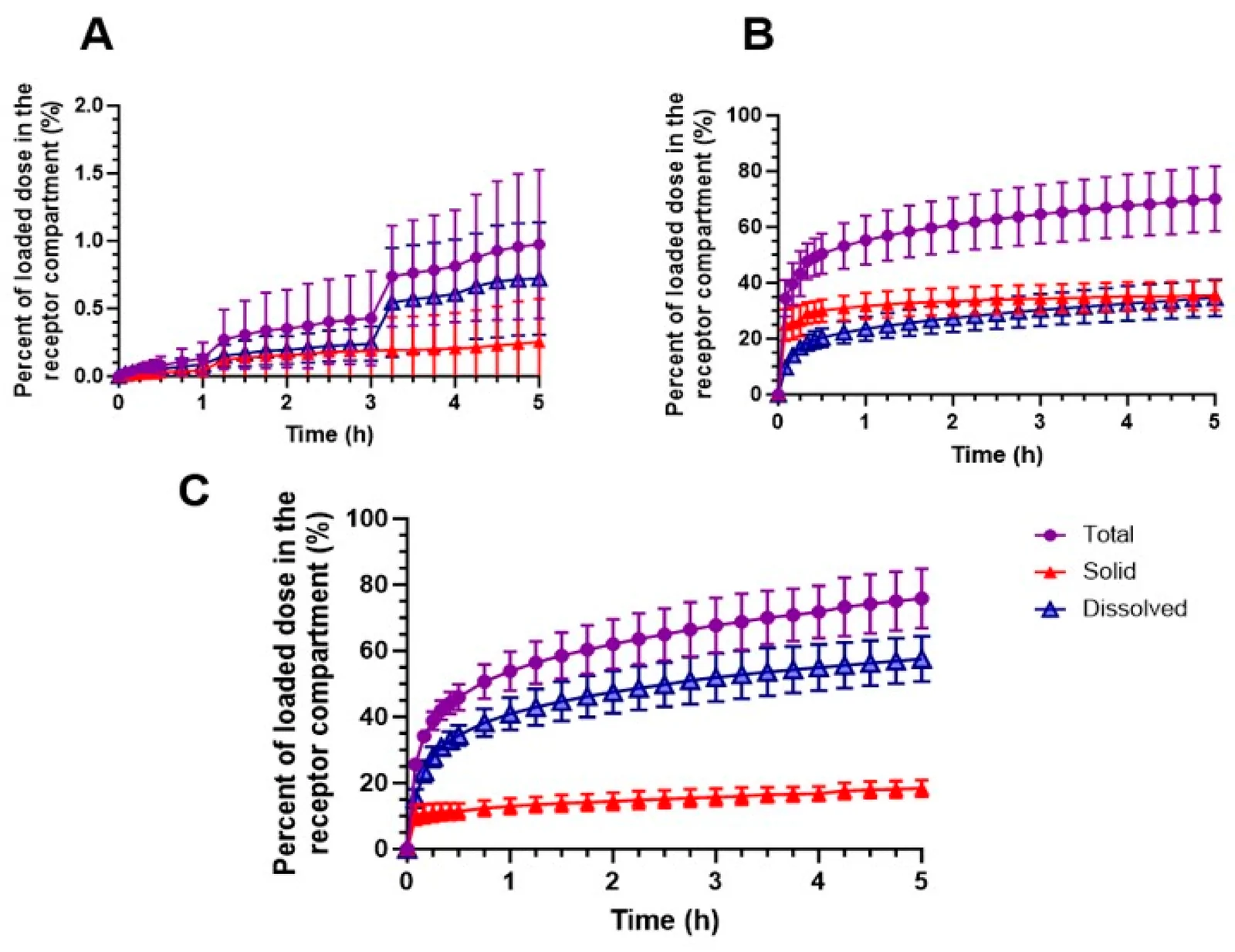
Dissolution profiles of OHet72 in SLF from (**A**) unprocessed drug, (**B**) OHet72 NCs, and (**C**) OHet72 nsMPs in terms of the total % of the loaded drug mass (dissolved and undissolved, purple lines) that reached the receptor compartment of the dissolution apparatus, and whether that drug mass was dissolved (drug content in the supernatant of samples collected at each time point, blue lines) or undissolved (drug content in the pellet of samples collected at each time point, red lines) (mean ± standard deviation, n = 3). Note differences in the Y axis of (**A**) compared to (**B**) and (**C**).

Figure 9 shows the dissolution profile of the unprocessed drug (9A), OHet72 NCs (9B), and OHet72 nsMPs (9C) over 5 h. Only 0.72 ± 0.34% of the loaded unprocessed drug dissolved over 5 h (Figure 9A, blue triangles). In addition, some of the smallest particles of unprocessed drug (0.25 ± 0.26% of loaded drug mass, red triangles) were able to pass through the filter sandwiched in the dissolution apparatus based on the analysis of the pellet recovered at each centrifugation timepoint. Thus, only 0.98 ± 0.26% of the loaded drug was detected in the receptor compartment, indicating that most of the unprocessed drug mass remained on the filter. In contrast, when the dissolution of OHet72 NCs was evaluated (Figure 9B), 70.12 ± 9.54% of the loaded drug mass was detected in the receptor compartment after 5 h, either as dissolved drug (34.65 ± 5.43% determined in the supernatant) or solid NCs (35.46 ± 4.26% determined in the pellet) that were able to go through the pores of the filter (0.45 µm) in the dissolution apparatus.

Evaluation of the dissolution of OHet72 nsMPs was comparable to that of OHet72 NCs, i.e., 75.89 ± 7.35% of the loaded drug was detected in the receptor compartment after 5 h (Figure 9C). However, in contrast to OHet72 NCs, the proportion of dissolved drug was higher (57.55 ± 5.60%) than the proportion of undissolved NCs that were able to go through the filter pores (18.34 ± 2.08%) in the dissolution apparatus.

**Figure 10:**
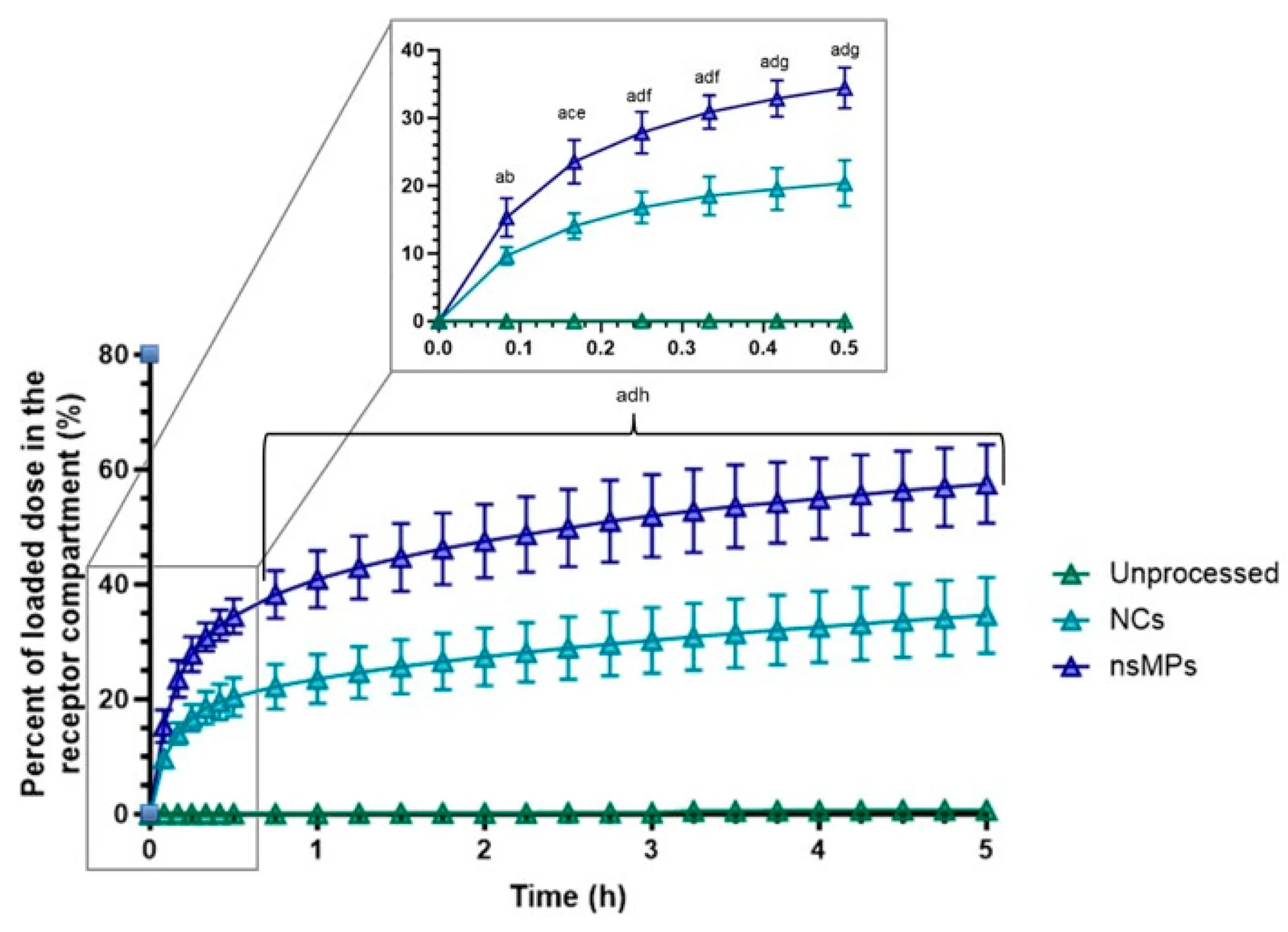
Cumulative percentage of OHet72 dissolved in SLF from unprocessed OHet72, OHet72 NCs, and OHet72 nsMPs (mean ± standard deviation, n = 3). Letters indicate significant differences between nsMPs and unprocessed drug (^a^ *p* < 0.0001), NCs and unprocessed (^b^ *p* < 0.05, ^c^ *p* < 0.001, ^d^ *p* < 0.0001) or nsMPs and NCs (^e^ *p* < 0.05, ^f^ *p* < 0.01, ^g^ *p* < 0.001, ^h^ *p* < 0.0001), determined by two-way ANOVA with multiple comparisons.

To compare the rate of OHet72 dissolution between formulations, Figure 10 shows only the individual plots of the dissolved drug determined in the receptor compartment of the dissolution apparatus.

Beginning at 5 min, the dissolution of OHet72 from NCs and nsMPs was significantly greater than that of the unprocessed drug (*p* < 0.05 and 0.0001 for NCs and nsMPs, respectively). After the first 10 min, the dissolution of OHet72 from nsMPs was significantly faster than that from the NCs (*p* < 0.05). Overall, the dissolution of OHet72 was 48 times faster when formulated as NCs compared to that of the unprocessed drug, whereas the dissolution of OHet72 was 79 times faster when formulated as nsMP compared to the unprocessed drug.

## 4. Discussion

Despite the introduction of two newer 6-month all-oral regimens to treat MDR-TB, the toxicity of the included drugs remains a significant drawback [36,37], and the need for effective drugs with less associated side effects remains a pressing need to reduce the occurrence of MDR-TB. OHet72 is a promising new drug candidate with activity against DS- and MDR-TB [11], but with limited aqueous solubility [12]. The present study reports the development of an optimized OHet72 NC formulation to enhance its dissolution, and its subsequent formulation into nsMPs to optimize their delivery to the alveoli, the main site of TB infection.

Some approaches that have been employed to enhance the dissolution of drugs with low water solubility include: (1) their physical transformation into the amorphous form; (2) formulation with excipients to aid dissolution, such as lipids or self-emulsifying agents; or (3) inclusion into nano-formulations such as liposomes or nanoparticles [38,39]. We previously reported the preparation of microparticles consisting of pure drug by spray drying [6], in which the drug transformed from the crystalline to the amorphous form. This physical transformation enhanced the apparent solubility of the drug 4-fold; however, within 2 h in water, the drug precipitated in its crystalline form, indicating that additional excipients such as polymers are required to stabilize the amorphous drug [40,41,42]. Elegant formulations such as the Pulmospheres^®^ microparticles have been used to deliver drugs with low water solubility, e.g., budesonide and amphotericin B, but the proportion of drug is small (3–50%) compared to that of the excipients [43,44]. Thus, depending on the therapeutic dose of the drug, it is likely that an inhaled dose of these formulations would be several-fold larger than that of a pure drug. In contrast, the advantages of preparing insoluble drugs as NCs consisting entirely of the drug itself, include enhanced dissolution rate, enhanced stability of the crystalline form and the potential of a small, inhaled dose.

**Figure 11:**
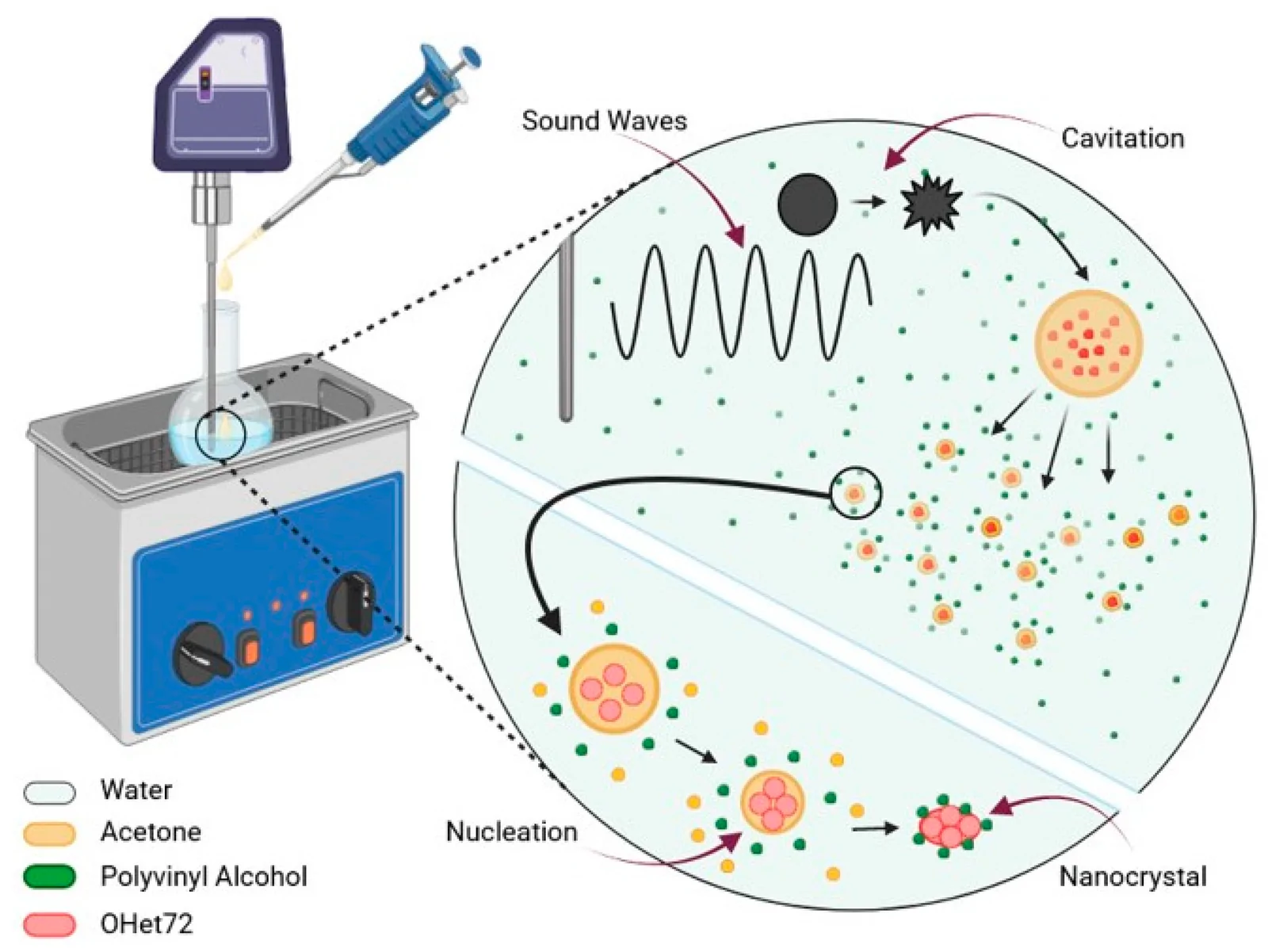
Representative diagram of the solvent–antisolvent crystallization method.

Initially, we optimized the characteristics of the OHet72 NCs by adjusting the sonication amplitude, sonication time, and ultrasonication time as input factors in DoE1, as these parameters have been reported to affect the size of crystals produced using the S-AS crystallization method [45]. To further reduce the size of the NCs, the effects of the S-AS ratio and the stabilizer concentration were evaluated in DoE2. Because the initial SEM evaluation of OHet72 NCs revealed an oblong morphology, the effects of these input parameters on the outcomes of NC length and width were evaluated separately. Statistical analysis of the parameters and outcomes in DoE1 determined that the width of NCs was mostly influenced by the interaction between sonication amplitude and sonication time, followed by ultrasonication time and then the interaction between the three parameters. These results are congruent with the sequence of the process to form NCs by the S-AS crystallization method (Figure 11): once the OHet72 saturated solvent solution is introduced into the antisolvent (2% PVA in water), mixing of the two phases is facilitated by the application of sound waves (A = sonication amplitude) provided by both probe sonication and an ultrasonicator water bath.

For OHet72 crystallization, the horn of the sonicator was placed in direct contact with the S-AS mixture to maximize the mixing energy transferred into the total volume of the solvent and antisolvent. Specifically, this mixing energy is produced by cavitation, a process in which sound waves are applied to a liquid (sonication amplitude) to cause localized pressure fluctuations. The pressure fluctuations, in turn, cause the formation and violent collapse of small vacuum bubbles, releasing high-speed jets, shockwaves, and shear forces into the liquid. In S-AS crystallization, these forces work to break up the droplets of saturated solvent within the bulk antisolvent into smaller units (Figure 11). Concurrently, the solvent diffuses into the antisolvent, increasing the localized supersaturation of the drug within each droplet. This process continues (sonication and ultrasonication times) until the degree of drug supersaturation reaches a critical point, and nucleation occurs, at which point the stabilizer in the antisolvent phase adsorbs onto the surface of the newly formed crystal to reduce subsequent crystal growth (Figure 11).

Specifically, sonication amplitude influences the frequency and intensity of cavitation within the S-AS mixture, affecting the size of the solvent droplets within the antisolvent and, thereby, the final size of the NCs. Sonication time influences the time available for drug nucleation and crystallization, meaning that a longer sonication time will favor the formation and crystallization of more NCs, while ultrasonication time influences the time available for crystal growth, and thus, a shorter time will prevent excessive NC growth. On the other hand, stabilizer concentration influences the mobility of the solvent droplets and the extent to which they break apart and disperse, whereas the S-AS ratio directly influences the volume available for the saturated solvent droplets to break up and disperse into. Thus, a 1% PVA allows more mobility of saturated solvent droplets, whereas a 1:20 S-AS ratio gives more space for these droplets to disperse, as described by the statistical analysis of the results of DoE2.

Kumar et al. [46] obtained oblong paclitaxel NCs with dimensions similar to ours using the S-AS method with ethanol as the solvent, water with 1% fucoidan as the antisolvent, a 1:10 S-AS ratio and a sonication amplitude of 50%; however, their sonication time was 10 min with a pulse cycle of 7 s on and 3 s off and without additional ultrasonication. In contrast, Pailla et al. [47] prepared zotepine NCs using the S-AS method with acetone as the solvent, water as the antisolvent, and a combination of Pluronic F-127, hydroxypropyl methylcellulose E15, and soy lecithin as stabilizers. By varying the sonication time from 5 to 20 min with ultrasound bursts set to 6 s on/3 s off with 40% amplitude, they obtained spherical nanoparticles with an average size of 519.26 ± 10.44 nm. Thus, it is evident that the size and shape of NCs prepared by the S-AS method are influenced by both the process parameters as well as the formulation ingredients.

A drawback of NC formulations is that NCs cannot be efficiently aerosolized as a dry powder form for pulmonary administration [48], they would need to be either incorporated into microparticles or suspended in a liquid for nebulization. We chose to prepare microparticles, and to do so, a suspension of OHet72 NCs in water was spray-dried with mannitol as the core since this method produces microparticles that are easily dispersible with high FPF while not decreasing the solubility of the loaded drug [6,49]. A similar formulation strategy was employed by Zhu et al. [50] to prepare nanocomposite microparticles containing ivacaftor nanoparticles within a core composed of colistin and leucine. In that case, the inclusion of the additional excipients resulted in the final ivacaftor nanocomposite microparticles containing only 2.8% ivacaftor, compared to 50% OHet72 in the nsMPs of the present study.

The physicochemical characteristics of the NCs and nsMPs were determined to confirm drug stability throughout the solvent–antisolvent and spray drying processes. FTIR analysis indicated that OHet72 remained chemically stable during both processes (Figure 5), whereas XRD analysis confirmed that OHet72 remained crystalline as both NCs and OHet72 nsMPs (Figure 6b). However, the peaks of the unprocessed drug and those of OHet72 NCs in the diffractogram were slightly different, suggesting that the drug in the NCs had a different polymorphic form than that of the unprocessed drug. This observation was supported by the slight shift in melting temperature of the OHet72 NCs versus the unprocessed drug shown in their DSC spectra as well as that of the OHet72 nsMPs (Figure 6a). It was also noted that the melting temperature in the DSC spectra of OHet72 in the nsMPs was slightly different than that of the NCs, likely due to physical interactions with mannitol, which was shown to have a much lower melting temperature. In a similar study, Leng et al. used a combination of XRD and DSC to confirm the stability of budesonide when preparing it as NCs and nanocomposite MPs [51]. Both the peaks in the diffractograms and the melting temperatures in the thermograms indicated that the drug remained crystalline throughout the spray drying process and no polymorphisms were present in the crystals. In contrast, XRD and DSC analyses of the zotepine nanoparticles prepared by Pailla et al. [47] revealed that the nanoparticles precipitated in an amorphous form, as confirmed by the characteristic “halo” pattern in the XRD chromatogram and both a shifted melting temperature and an exothermic phase transition peak in the DSC thermogram.

The aerosol performance of OHet72 formulations was evaluated as wet aerosols generated with the PARI LC Star nebulizer (from NC suspended in saline) and as dry aerosols generated with an DPI (from nsMPs). The dose of OHet72 emitted from the nebulizer was 17 times smaller (0.189 ± 0.071 mg) than that emitted from the DPI (3.20 ± 0.51 mg). In part, this difference occurs because the nebulizer was only operated for 5 min to prevent overloading of the NGI stages [52,53]. Both wet and dry aerosols had similar MMADs (3.13 ± 0.43 and 3.80 ± 0.49 µm, respectively) and GSDs (2.81 ± 0.18 and 2.60 ± 0.32, respectively), but the FPF of wet aerosols was significantly larger compared to dry aerosols (54.63 ± 5.83 vs. 37.16 ± 6.67%, *p* = 0.0269). This difference can be observed from the NGI deposition profile for both wet and dry aerosols, as the proportion of coarse aerosols is significantly larger when generated from the nsMPs than from the NC suspension, whereas the proportion of fine and extra-fine aerosols is larger when generated from the NC suspension. This may be due to possible agglomeration of the nsMPs while being aerosolized with a limited volume of air, whereas the larger volume of air supplied to the nebulizer to generate the aerosols aids in their dispersion. Even though the proportion of fine and extra-fine aerosols as well as the FPF are larger for aerosols generated by the nebulizer, when the emitted dose is considered, it is evident that inhalation of OHet72 nsMP would achieve a larger dose deposited in the alveoli, highlighting the advantages of the nsMPs over the NCs.

After drug deposition, it is generally assumed that the drug must dissolve before it can be absorbed into the lung tissue [54,55,56,57]. As expected, the apparent solubility of the NCs was 14 times higher than that of the unprocessed drug, mainly due to the size of the NCs, which were approximately 1000× smaller than the unprocessed drug, resulting in a significant increase in surface area and corresponding enhanced solubility. It is also likely that the different crystalline polymorphic form of the NCs produced in the present study (Figure 6b) contributes to this enhanced solubility. Based on the thermal analysis of the formulations (Figure 6a), the OHet72 NCs had a slightly lower melting temperature compared to the unprocessed drug (202.71 °C vs. 205.28 °C, Figure 6a), which could correlate with an increase in solubility. Moreover, the apparent solubility of the nsMPs was 15 times higher than that of the unprocessed drug and 1.1 times greater than that of the NCs. Given that the crystallinity of the NC polymorph was preserved throughout the spray drying process, the increase in apparent solubility of the nsMPs compared to the NCs may be explained by the lower OHet72 melting temperature in the nsMPs compared to the NCs (197.35 °C vs. 202.28 °C, Figure 6a), possibly due to molecular interactions between OHet72 and mannitol. Similar to the present study, Duret et al. [58] developed itraconazole nanocomposite microparticles that exhibited nearly a 10-fold increase in saturation solubility in simulated lung fluid compared to the unprocessed drug. In contrast, the apparent solubility of tadalafil NCs prepared by Rad et al. increased only 3.46 times in water and 2.78 times in PBS [59]. The difference in the enhancement of apparent solubility between the tadalafil NCs and OHet72 NCs may be attributed to the magnitude of particle size reduction corresponding to the increase in surface area in these cases. While OHet72 NCs were approximately 1000 times smaller than the unprocessed drug, tadalafil NCs were less than 200 times smaller than the unprocessed drug.

Consistent with the enhanced apparent solubility, an initial drug dissolution “burst” was observed for the NC and nsMP during the first five minutes of the study, most likely due to the passage of drug particles smaller than 450 nm through the 0.45 µm pores of the glass fiber filter. This was followed by a zero-order, constant dissolution rate until approximately 30 min, possibly due to faster drug dissolution of those NCs slightly larger than 450 nm leading to their subsequent reduction in size which would then facilitate their passage through the pores of the glass fiber filter. The OHet72 in both the NCs and the nsMPs exhibited enhanced dissolution compared to the unprocessed drug (Figure 11). The extent of drug dissolution in NCs was 48× that of the unprocessed drug, whereas for nsMPs it was 78× that of the unprocessed drug. The greater drug dissolution in nsMPs may be explained by the dispersion of NCs within the mannitol matrix in the nsMPs, which could facilitate the wetting of NCs and reduce their aggregation when introduced into media.

The advantage of formulating drugs into NCs to enhance drug dissolution has also been reported by others, including Zhang et al., who described complete dissolution of baicalein NCs within 15 min compared to only 29.1% of the unprocessed drug over 60 min [60]. Likewise, Rad et al. reported that dissolution of tadalafil from NCs was 3.45 times higher after 90 min compared to the unprocessed drug [59], and Yu et al. determined that the dissolution of ivacaftor from nanocomposite microparticles was 3.29 times higher after 6 h compared to a physical mixture of the individual components [61]. Herein, the dissolution of OHet72 NCs and nsMPs increased by 48 and 7 times, respectively, after 5 h.

## 5. Conclusions

The size of OHet72 NCs was optimized to maximize their incorporation into nsMPs, and the size of the resulting OHet72 nsMPs was also optimized to be suitable for alveolar deposition (MMAD ~3 µm). Most importantly, the apparent solubility of the drug in both NCs and nsMPs and their dissolution rates in SLF were significantly higher than those of the unprocessed drug. Together, these data suggest that pulmonary delivery of a suitable dose of OHet72 NCs or nsMPs is likely to result in sufficient dissolution in the lung environment to achieve theoretical therapeutic concentrations in the lungs (MIC = 1 µg/mL), given the appropriate dosing regimen. Future studies will determine the in vivo pharmacokinetic parameters that describe the disposition of OHet72 after pulmonary administration of nsMPs and will inform the theoretical therapeutic dose to be used in efficacy studies using these formulations.

## Figures and Tables

**Table 1:** Solvent–antisolvent crystallization parameters in DoE1 to optimize the sizes of OHet72 NCs, the resulting diameters (length and width), and the corresponding GSDs.

Run	Sonication Amplitude (%)	Sonication Time (min)	Ultrasonication Time (min)	Dg Width (nm)	GSD_Dg_ Width	Dg Length (nm)	GSD_Dg_ Length
1	50	30	30	760	2.763	1500	3.000
2	100	60	60	480	3.021	1050	2.238
3	50	60	60	1500	2.933	2000	2.850
4	50	60	30	700	1.440	1300	1.750
5	50	30	60	750	2.070	1550	1.740
6	100	30	30	760	1.776	1400	2.107
7	100	60	30	500	3.100	1100	2.682
8	100	30	60	860	2.070	1160	1.940

**Table 2:** Solvent–antisolvent crystallization parameters in DoE2 to further reduce the sizes of OHet72 NCs, the resulting diameters (length and width), and their corresponding GSDs.

Run	PVA Concentration (% *w*/*v*)	Solvent–Antisolvent Ratio	Dg Width (nm)	GSD_Dg_ Width	Dg Length (nm)	GSD_Dg_ Length
1	2	1:20	210	1.67	410	1.71
2	1	1:10	215	1.71	410	2.04
3	2	1:10	285	1.93	595	2.21
4	1	1:20	145	1.90	255	1.94

## Data Availability

All the relevant data are available from the corresponding author upon request.

## Abbreviations

The following abbreviations are used in this manuscript:

D_ae_	Aerodynamic Diameter
Dg	Geometric Diameter
Dv	Volume Diameter
DoE	Design of Experiments
DSC	Differential Scanning Calorimetry
DPI	Dry Powder Inhaler
FPF	Fine Particle Fraction
FTIR	Fourier Transform Infrared Spectroscopy
GSD	Geometric Standard Deviation
ISM	Impactor Sized Mass
MMAD	Mass Median Aerodynamic Diameter
nsMPs	Nanostructured microparticles
NCs	Nanocrystals
NGI	Next Generation Impactor
PVA	Polyvinyl alcohol
RCF	Relative Centrifugal Force
RPM	Revolutions Per Minute
S-AS	Solvent–Antisolvent
SEM	Scanning Electron Microscopy
SLF	Simulated Lung Fluid
TB	Tuberculosis
UV	Ultra-violet
XRD	X-ray Diffraction
